# The domain-general and domain-specific cognitive profiles in high and low-achieving Chinese L2 learners

**DOI:** 10.3389/fpsyg.2025.1577986

**Published:** 2025-10-09

**Authors:** Qing Xiao, Yue Shi, Dazhi Cheng

**Affiliations:** ^1^Department of Language Teaching and Research, Beijing Chinese Language and Culture College, Beijing, China; ^2^School of Psychology, Capital Normal University, Beijing, China

**Keywords:** Chinese, second language, cognitive abilities, phonological processing, morphological awareness

## Abstract

Although both general cognitive and domain-specific skills are important for Chinese L2 learning, it remains unclear whether high and low-achieving Chinese L2 learners differ in specific cognitive abilities. This study examines both domain-general and domain-specific cognitive profiles in high and low-achieving Chinese L2 learners. Sixty-four Chinese L2 learners from Southeast Asia were categorized into high-achieving group and low-achieving group based on academic performance and teacher evaluations. The study assessed general cognitive functions, including working memory, attention, and phonological processing, as well as domain-specific skills such as morphological awareness. The results showed that low-achieving Chinese L2 learners performed poorly in phonological processing tasks such as verbal working memory and pitch matching. They also underperform in morphological awareness tasks such as nonword identification and homophone judgment. These findings demonstrated the critical role of both phonological processing and morphological awareness in Chinese L2 learning suggesting potential interventions targeting these cognitive areas to improve learning outcomes for low-achieving Chinese L2 learners.

## Introduction

The Chinese language widely used both within and outside of China, has witnessed a remarkable increase in the number of learners worldwide (Gong et al., 2020; Xiong and Peng, 2022). By the end of 2023, the number of people outside China learning Chinese as a second/foreign language (i.e., L2) had exceeded 30 million, while the cumulative number of Chinese language learners and users globally surpassed 200 million (Ministry of Education of the People's Republic of China, 2024).

The theoretical hypothesis for understanding the cognitive processing involved in second language (L2) acquisition was called Fundamental Difference Hypothesis (Bley-Vroman, 1989). It proposed that first language (L1) and L2 acquisition had differentially cognitive mechanisms. While L1 acquisition is primarily guided by Universal Grammar and an innate language acquisition device, L2 acquisition depends largely on general cognitive abilities, such as memory, attention, and problem-solving. This distinction between L1 and L2 acquisition provides a crucial framework for understanding the cognitive demands of learning a second language, especially in linguistically complex languages such as Chinese (Kanno, 1997).

Cross-linguistic studies further emphasize the unique cognitive demands for Chinese L2 learners. Compared to alphabetic languages, the Chinese writing system combined semantic radicals and phonetic components requiring the simultaneous processing of visual, phonological, and morphological information (Guan et al., 2014; Ip et al., 2019). For Chinese language, each character represents a morpheme rather than a phoneme. In contrasts, alphabetic characters map directly to phonemes (Liu and McBride-Chang, 2010; McBride C. A., 2016; Xia et al., 2023). These structural differences not only demand increased working memory capacity for character recognition and reading comprehension, but also highlight the critical role of morphological awareness (MA) and phonological processing in Chinese L2 learning. For instance, Ruan et al. (2018) demonstrated that MA accounted for a significantly higher proportion of variance in Chinese reading comprehension (15.2%) compared to alphabetic languages (10.2%). Taken together, the Fundamental Difference Hypothesis and cross-linguistic perspectives emphasize the important role of domain-general and domain-specific cognitive abilities in Chinese L2 learning.

### The role of general cognitive abilities in Chinese L2 learning

General cognitive abilities, such as working memory, attention, and processing speed, play a critical role in second language (L2) learning (Frischkorn et al., 2019; Kormos, 2012; Linck et al., 2014; Luque and Morgan-Short, 2021). Working memory referred to the ability that temporarily hold and manipulate information in verbal and visuospatial domains. It is particularly crucial for managing the complex linguistic tasks inherent in L2 acquisition (Schwieter et al., 2022). Most previous studies on working memory in L2 word learning have found that working memory is especially important when learning new characters (Baddeley et al., 1998; Shen and Park, 2020; Wen and Li, 2019). As Chinese character represents a morpheme, L2 learners must remember both visual configuration and phonetic and semantic cues (Wen et al., 2015).

Working memory was also important for foreign language learning (Juffs and Harrington, 2011; Szmalec et al., 2013). For example, Verhagen and Leseman (2016) found that verbal working memory contributes significantly to L2 grammar learning, especially in tasks involving complex sentence structures. Similarly, previous studies demonstrated that verbal working memory facilitates speech production such as monitoring grammatical accuracy and maintaining discourse coherence (Juffs and Harrington, 2011; Martin and Ellis, 2012). Although the importance of verbal working memory in L2 learning is established, the role of spatial working memory in L2 learning remains debated. Ho et al. (1999) reported that spatial working memory was significant in reading Chinese. However, Kim et al. (2015) found that verbal working memory plays a more important role than spatial working memory even in learning to read ordinary.

Beyond working memory, attention is a strong predictor of success in both L2 grammar learning and vocabulary acquisition (Robinson et al., 2012). In the context of Chinese, where learners need to process complex phonological, orthographic, and semantic information simultaneously, attention becomes crucial. In particular, the ability to suppress irrelevant stimuli and focus on the linguistic aspects of characters, tones, and sentence structures is fundamental for mastering Chinese as an L2 (Darcy et al., 2014; Ghaffarvand Mokari and Werner, 2019).

Another important factor is processing speed, or the rate at which individuals can perform cognitive tasks. Faster processing speed has been linked to better L2 performance, especially in tasks that require quick retrieval of vocabulary and real-time speech production (Zhang and Yang, 2023). For Chinese language learning, learners should quickly recognize and produce tonal and character-based linguistic features that were impacted by processing speed (Luque and Morgan-Short, 2021; Segalowitz and Frenkiel-Fishman, 2005).

In addition to these core constructs, other cognitive abilities such as non-verbal IQ and cognitive flexibility may also contribute to L2 learning outcomes, especially when learners engage in novel or complex tasks (Li, 2015; Mayora, 2010). Therefore, to comprehensively assess the cognitive underpinnings of Chinese L2 word learning, the current study employed nine tasks that tap into a broad range of cognitive functions.

### The domain-specific cognitive profile on Chinese L2 learning

Morphological awareness (MA), defined as the ability to recognize and manipulate the morphemic structure of words, is a critical cognitive skill for Chinese L2 learners (McBride C. A., 2016; Pang and Son, 2024). This cognitive ability plays a unique role in Chinese learning, which differs greatly from alphabetic-phonemic languages where word recognition is based on letter recognition. Unlike alphabetic languages, Chinese belonging to a morphosyllabic script lacks consistent correspondence rules (McBride C., 2016). Consequently, morphological awareness becomes crucial, as learners must rely on the morphemic structure of characters rather than phoneme-grapheme mappings.

Moreover, the high frequency of homophones in Chinese [e.g., 和/he4/(together) and河/he4/(river)], combined with the predominance of compound words, makes morphological awareness especially salient for reading comprehension (Liu et al., 2013; McBride-Chang et al., 2008). Numerous studies have suggested that morphological awareness distinctly supports Chinese reading (Chen, 2021; Ke, 2025; Liu et al., 2024; Zhang et al., 2022). For instance, Chen (2021) demonstrated that morphological awareness was significantly positively correlated with Chinese word reading, which is consistent with prior meta-analyses conducted by Ruan et al. (2018), showing correlations of 0.385 with Chinese reading fluency and 0.393 with Chinese reading accuracy.

### The current study

To sum up, existing research has identified the important role of cognitive abilities in L2 learning. Despite the acknowledged importance of both general cognitive and domain-specific skills in Chinese L2 learning, it remains unclear whether high and low-achieving Chinese L2 learners differ in specific cognitive abilities (Siok and Tan, 2022). Much of the existing literature has focused on alphabetic languages such as English (Chen, 2021; Chen et al., 2022; Xie et al., 2021). Although morphological awareness has been identified as a key literacy skill in native Chinese speakers (Liu et al., 2013), whether there are morphological awareness differences between high- and low-achieving Chinese L2 learners is still unclear. Thus, there is a notable gap in comparative studies focusing on cognitive differences between high- and low-achieving Chinese L2 learners.

Therefore, the present study aims to examine the specific cognitive profiles of domain-general and domain-specific skills in high and low-achieving Chinese L2 learners. This study focuses on two research questions: (1) Do high- and low-achieving Chinese L2 learners differ in their domain-general and domain-specific cognitive abilities? (2) To what extent do high- and low-achieving Chinese L2 learners differ in domain-specific cognitive skills? High and low-achieving Chinese L2 learners were both selected and received a series of cognitive ability assessments. It is hypothesized that low-achieving students of Chinese L2 learners will demonstrate underperformance‌ in both general cognitive abilities and domain-specific skills. These findings would provide insights into the cognitive mechanisms underlying these learners’ difficulties and imply future interventions aimed at improving their cognitive abilities.

## Methods

### Participants

Sixty-four undergraduate learners of Chinese were selected from 175 students in first-year college from Southeast Asia (Thailand and Indonesia). All participants had normal or corrected-to-normal vision. They are students of Chinese Education from a university in Beijing. Before they learn Chinese, none of the participants had prior experience in L2 courses. By the time the study was conducted, the participants had learned Chinese as their second foreign language for half a year in high intensity (22 class hours per week). Based on their performance of placement tests including written and oral problems and teachers’ evaluations, they were divided into two distinct proficiency groups: high-achieving and low-achieving learners.

High-achieving Chinese L2 learners were those who ranked in the top 20% of Chinese achievement tests. Meanwhile, class teachers evaluated that these students achieved the top five in the class rankings (including the parallel ranking of the class) in each of the two-quarters of the first-year Chinese course. In total, 34 high-achieving students participated in this experiment, including 6 males and 28 females (ages ranging from 17 years to 21 years; SD = 0.98).

Low-achieving Chinese L2 learners were those who ranked in the bottom 20% of Chinese achievement test. Meanwhile, class teachers evaluated that these students achieved the bottom five in the class rankings (including the parallel ranking of the class) in each of the two-quarters of the first-year Chinese course. In total, 30 low-achieving students participated in this experiment, including 10 males and 20 females (ages ranging from 17 years to 22 years; SD = 1.32). There were no significant differences between high- and low-achieving group in age (*t* = 0.55, *p* = 0.59) and gender (χ2 = 2.09, *p* = 0.15).

### Tasks

#### Chinese achievement test

The teaching and research section in the university developed a general Chinese achievement test for all students at the end of each semester. Chinese achievement test consists of Chinese character writing, word identification, grammar structure, sentence and text comprehension, and composition. In Chinese achievement tests, teachers evaluate students’ mastery of Chinese knowledge learned during the semester, including basic knowledge such as Chinese characters and words, comprehension ability, and comprehensive application ability. Chinese achievement test is scored out of 100 points: 10 points for Chinese character writing, 30 points for word comprehension and application, 20 points for grammar, 20 points for sentence and text comprehension, and 20 points for composition. Chinese achievement test lasted for 90 min.

#### Domain-general cognitive abilities

**Figure 1:**
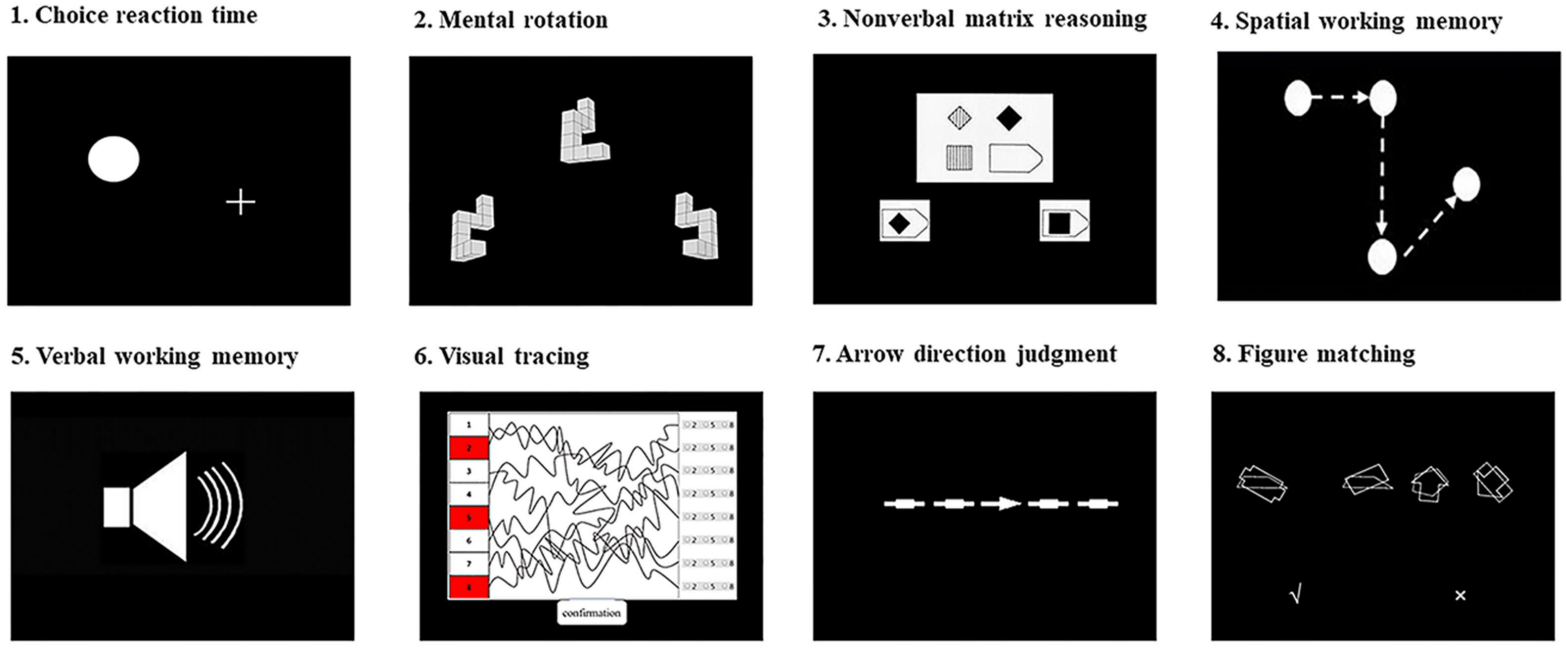
Schematic representation of tests used in the study.

A total of 9 tests were used by web-based applications in the “Online Psychological Experimental System (OPES)”.^1^ For most tests, the participants responded to two-choice options by pressing the Q and P keys on a computer keyboard to choose the correct answers. The other response modes are explained below in details. For each test, the split-half reliabilities were calculated. All tasks have shown acceptable reliabilities, ranging from 0.86 to 0.96. The schematic representations of tests are displayed in Figure 1. The tests are introduced as follows.

##### Choice reaction time

A basic reaction time task was used to account for the influence of manual response and mental processing speed [similar to the reaction time task included in Butterworth (2003)]. In each trial of the choice reaction time test, a white dot was presented on a black screen, either to the left or to the right of a fixation cross. The position of the dot was within 15° visual angle from the cross. Participants were asked to press the “Q” key if the dot appeared on the left and the “P” key if it appeared on the right. There were 30 trials in total (15 trials with the dot on the left and 15 trials with the dot on the right). The size of the screen on which the dot appeared varied randomly across trials. Interstimulus intervals varied randomly between 1,500 ms and 3,000 ms. This test had excellent internal consistency, with Cronbach’s alpha of 0.96.

##### Mental rotation

The mental rotation task was adapted from a three-dimensional image matching task (Vandenberg and Kuse, 1978), which was used to assess visuospatial ability. In each trial, a three-dimensional image was presented on the upper part of the screen, and two more were presented on the lower part of the screen. Participants were asked to choose which image from the lower part of the screen matched the image on the upper part; the matching image could be identified only by mental rotation. The non-matching image was a rotated mirror image of the target. The rotation angles of the matching images ranged from 15° to 345°, in intervals of 15°. Participants pressed the “Q” key to choose the image on the left and the “P” key to choose the image on the right. The stimuli remained on the screen until the participant responded by pressing the “P” or the “Q” key. The mental rotation test consisted of 180 trials. This was a time-limited (3 min) test. Cronbach’s alpha of split-half reliability was 0.87.

##### Non-verbal matrix reasoning

A simplified version of Raven’s Progressive Matrices test (Raven, 1998) was used to assess general intelligence and inductive reasoning ability. In this task, participants had to identify a missing segment that would complete a figure’s pattern. Two candidate answers were presented side-by-side beneath each problem; participants were instructed to press “Q” if the missing segment was on the left and “P” if it was on the right. The test consisted of 80 trials. This was a time-limited (3 min) test. This test had an internal consistency with Cronbach’s alpha of 0.84.

##### Spatial working memory

This task was similar to the Corsi block task (Corsi, 1973). Dots were sequentially presented in an implicit lattice of 3 by 3 on the computer screen. Each dot was presented for 1,000 ms, and dots were presented with an interval of 1,000 ms. After the last dot was presented and disappeared, the participants clicked the positions where the dots had appeared in the same sequence as their appearance. The number of dots ranged from 4 to 9. There was no feedback from participants. The average distance between the position where the dot appeared and the position where participants clicked was calculated and treated as an index of spatial working memory. Larger average distances reflected poorer spatial working memory. This test had an internal consistency with Cronbach’s alpha of 0.92.

##### Verbal working memory

The forward and backward digit span task was used to evaluate verbal working memory ability, which was adapted from the Wechsler Intelligence Scale (Wechsler, 1974). To test the participants’ forward and backward memory span, we presented a series of digits aurally. The sound duration for each digit was standardized to 200 ms. Participants were asked to remember the order of the digits and report them at the end of each series. The test began with three items (digits) for the forward and two items for the backward digit span. The number of items increased gradually until the participants failed to report them correctly for three consecutive trials. This test had an internal consistency with Cronbach’s alpha of 0.95.

##### Visual tracing

The task was adapted from Groffman’s visual-tracing test to examine visual attention (Groffman, 1966). Several curved lines within a square interweaved with one another starting from the left side of the square and ending on the right side. Participants were asked to track a particular line from the beginning to the end using only their vision (i.e., they were not allowed to use a finger or the cursor or an object to trace) and then to mark the correct endpoint. This task became more difficult as the total number of lines increased. There were 12 pictures, each used in 3 trials. This was a time-limited (4 min) task. This test had an internal consistency with Cronbach’s alpha of 0.92.

##### Arrow direction judgment

The attention test was adopted from Fan et al.’s attention network test (Fan et al., 2002). It has been extensively used to assess attention (Matthews et al., 2010; Rueda and Posner, 2013; Weaver et al., 2009). Five arrows in one line were presented on the screen, and the participants needed to judge the direction of the arrow in the middle by pressing the left or the right key to be consistent with the direction of the arrow. Before the arrow line was presented, there was a cue for alerting. There were two types of middle arrows: Their direction was either the same as that of the other arrows (i.e., the congruent condition) or opposite of the direction of the other arrows (i.e., the incongruent condition). There were 192 trials presented in two blocks. Before each trial, a “+” sign was presented for a random duration between 400 ms and 1,200 ms, followed by the cue sign for 100 ms, then the “+” sign again for 400 ms, and finally by the arrow line. The arrow line was presented for 1,700 ms or until the participants pressed a key. The average percentage of correct responses was 98.2%, so we just used the reaction time on the correct trials as the dependent variable. This test had an internal consistency with Cronbach’s alpha of 0.96.

##### Figure matching

The figure-matching task was adapted from an identical picture test in the Manual for Kit of Factor-Referenced Cognitive Tests (Ekstrom et al., 1976). It was used to assess visual perception. There were 120 trials, each containing one target picture on the left side and three candidate pictures on the right side (see Figure 1). The pictures were constructed from 150 abstract line figures. The four pictures were presented simultaneously for 400 ms. Participants were asked to judge whether the picture on the left side also appeared on the right side, by pressing the button “Q” for yes or “P” for no. The 120 trials were grouped into three 40-trial sessions. Children were asked to complete all trials. This test had an internal consistency with Cronbach’s alpha of 0.86.

##### Pitch matching

This task was adapted from the same-different tone task to assess phonological identification (Tallal and Piercy, 1973). There were totally fifteen 100 ms complex tones of frequencies within the speech range (fundamental frequency: Tone 1 = 100 Hz, Tone 2 = 150 Hz, Tone 3 = 250 Hz, Tone 4 = 300 Hz, Tone 5 = 350 Hz, Tone 6 = 400 Hz, Tone 7 = 450 Hz, Tone 8 = 500 Hz, Tone 9 = 600 Hz, Tone 10 = 650 Hz, Tone 11 = 700 Hz, Tone 12 = 800 Hz, Tone 13 = 850 Hz, Tone 14 = 900 Hz, Tone 15 = 1,500 Hz, rise/fall time 40 μs^−1^). Three pitches were presented sequentially. Participants were asked to judge which one of the first two pitches was the same as the third pitch. There were 120 trials for this test and they appeared on the screen in a pseudo-random order. This test had an internal consistency with Cronbach’s alpha of 0.89.

#### Domain-specific cognitive abilities

Three tasks (i.e., nonword identification, morpheme judgment and homophone judgment) for domain-specific cognitive abilities were conducted to evaluate morphological awareness.

##### Nonword identification

This test was adapted from a nonword repetition task (Shu et al., 2006). It was used to assess orthographic skills. In each trial, participants were asked to judge whether the symbol presented on the paper was a real Chinese character. There were three conditions for the symbols: real Chinese words that the participants never learned before; pseudowords that accord with the constituting rules of Chinese characters; and nonwords that break the constituting rules of Chinese characters. This task consisted of 144 trials, each condition has 48 trials. This test had an internal consistency with Cronbach’s alpha of 0.75.

##### Morpheme judgment

This task was adapted from the morpheme judgment test (Shu et al., 2006). Task stimuli were selected from Chinese textbooks for foreigners at the primary level. For each trial, a target Chinese character in the two-character word was presented, and two candidate words were presented on the right side. Participants needed to select one of two candidate words to match the meaning of the target Chinese character [e.g., a shared syllable *bao4* in *kan4bao4* (meaning read newspaper) and *bao4zhi3* (meaning newspaper)], not *bao4ming2* (meaning sign up). There were 20 trials in total. This test had an internal consistency with Cronbach’s alpha of 0.66.

##### Homophone judgment

Homophone judgment tasks both assess phonological skill and morphological awareness (Shu et al., 2006). In this task, two-paired morpheme Chinese characters were presented to the participants (e.g., *bu4* [meaning no] and *bu4* [meaning step]). In this example, two paired characters shared the same sound (homophone/homograph). Participants were asked to judge whether the two characters had the same pronunciation or not. There were 40 items in total, in which half of the stimuli had the same morpheme and the other half of the stimuli had a different morpheme. This test had an internal consistency with Cronbach’s alpha of 0.71.

### Research procedures

The battery of domain-general cognitive tasks was administered in two 45 min sessions. The testing was conducted in two groups (one group at a time) in computer classrooms. Each class was monitored by four experimenters, as well as one of the teachers of that class. The tasks were administered in the same order for all participants. For each task, an instruction was given and a practice session was completed before the formal testing. The practice session for each task consisted of either four or six trials, which were similar to those used in the formal testing. The computer provided the participant with feedback on the screen after each practice trial. Participants could ask experimenters any questions that they had during the practice session. After all participants had finished the practice session and had no more questions, they were asked to press any key to begin the formal testing.

For all but one task, the participants responded by pressing one of two keys (“P” or “Q”) on a computer keyboard. Only for the visual tracing task, they marked the correct endpoint after tracing a particular line. Participants” responses were automatically recorded by the computer.

Domain-specific cognitive tasks for morphological awareness were conducted by paper and pencil tests. The correct response of participants was recorded and analyzed.

### Statistical analyses

For all but one task (i.e., choice reaction time) in domain-general cognitive abilities, we calculated corrected scores by subtracting the number of incorrect responses from the number of correct responses to control for the effect of guessing (Cirino, 2011; Hedden and Yoon, 2006; Salthouse and Meinz, 1995). For the choice reaction time, only the median reaction time for each participant was calculated. Their mean error rate was low (4.4%), and thus was not further analyzed. Independent samples *t*-test compared performance on all tests between high and low-achieving Chinese L2 group. In addition, correlation analyses were performed to investigate the relationships between cognitive measures.

## Results and discussion

### Descriptive statistics and Levene’s test

Table 1 displays the mean scores and standard deviations for all cognitive tasks for both the high-achieving and low-achieving groups. Prior to reporting independent samples *t*-tests results, assumption of homogeneity of variance was assessed using Levene’s test. For variables where the assumption was violated (*p ≤* 0.05), the *t*-tests results for equal variances not assumed are reported. For variables where the assumption was true (*p >* 0.05), the *t*-tests results for equal variances assumed are reported.

**Table 1:** The cognitive performance of high-achieving and low-achieving students in Chinese second language learning (mean with standard deviation).

Tasks	High**-**achieving students	Low-achieving students	*t* value
Chinese achievement	94.04 (4.75)	60.28 (17.78)	10.09***
Choice reaction time	393.29 (82.92)	382.43 (68.83)	0.57
Mental rotation	21.88 (7.96)	18.87 (9.59)	1.37
Non-verbal matrix reasoning	20.79 (9.14)	18.17 (8.24)	1.20
Spatial working memory	5.68 (1.53)	5.83 (1.18)	−0.46
Visual tracing	15.53 (6.19)	17.23 (6.03)	−1.13
Figure matching	73.94 (20.46)	64.67 (24.60)	1.65
Pitch matching	47.18 (29.09)	32.87 (27.76)	2.00*
Working memory			
Verbal working memory (Forward)	5.94 (1.92)	4.80 (2.01)	2.32**
Verbal working memory (Backward)	5.26 (1.02)	4.03 (2.48)	2.53**
Arrow direction judgment	46.18 (4.30)	42.93 (17.02)	1.06
Nonword identification	0.95 (0.06)	0.89 (0.10)	1.24
Morpheme judgment	0.92 (0.07)	0.81 (0.14)	3.80***
Homophone judgment	0.88 (0.08)	0.67 (0.11)	8.73***

* *p* < 0.05, ** *p* < 0.01, *** *p* < 0.001.

### Differential roles of general cognitive abilities in Chinese L2 learning

The independent samples *t*-test showed that the high-achieving group (*M* = 94.04, SD = 4.75) had better performance compared to the low-achieving group (*M* = 60.28, SD = 17.78) in Chinese achievement, *t* = 10.09, *p* < 0.001. In terms of phonological processing, the high-achieving group (*M* = 47.18, SD = 29.09) showed better performance compared to the low-achieving group (*M* = 32.87, SD = 27.76) on pitch matching, and this difference was statistically significant, *t* = 2.01, *p* < 0.05. Mandarin Chinese is a tonal language in which lexical meaning is encoded by pitch patterns. Precise perception and reproduction of tone contours are foundational for accurate word recognition and pronunciation (Wang et al., 2003). Enhanced pitch matching likely enabled high-achievers to form more robust mappings between auditory input and orthographic representations, facilitating both listening comprehension and character acquisition (Chen et al., 2023). Moreover, high-achieving learners (*M* = 5.94, SD = 1.92) demonstrated significantly greater verbal working memory capacity than low-achieving learners (*M* = 4.80, SD = 2.01) in both forward digit span, *t* = 2.32, *p* < 0.05, and backward digit span (high-achieving: *M* = 5.26, SD = 1.02; low-achieving: *M* = 4.03, SD = 2.48), *t* = 2.53, *p* < 0.05. This finding indicated that constrained verbal memory reduces the efficiency of lexical retrieval and syntactic integration (Perfetti, 2007). Chinese character represents a bound morpheme with complex phonological and semantic associations, in which high VWM capacity is especially critical for vocabulary acquisition, sentence processing, and reading comprehension (Table 2).

**Table 2:** Intercorrelations among task scores for all groups.

Tasks	1	2	3	4	5	6	7	8	9	10	11	12	13
1. Chinese achievement	–												
2. Choice reaction time	0.16	–											
3. Mental rotation	0.12	−0.05	–										
4. Non-verbal matrix reasoning	0.32*	0.07	0.32*	–									
5. Spatial working memory	−0.05	−0.15	0.15	0.08	–								
6. Visual tracing	−0.04	−0.011	0.22	0.23	0.51**	–							
7. Figure matching	0.36**	−0.07	0.17	0.24	0.29*	0.271*	–						
8. Pitch matching	0.18	0.05	0.27*	0.07	0.10	0.00	0.12	–					
9. Forward digit span	0.28*	−0.09	0.44**	0.17	0.13	0.09	0.32**	0.11	–				
10. Backward digit span	0.44**	−0.04	0.34**	0.38**	0.22	0.18	0.27*	0.13	0.31*	–			
11. Arrow direction judgment	0.09	−0.03	0.21	0.13	−0.04	0.02	−0.08	−0.03	0.07	0.07	–		
12. Nonword identification	0.45**	0.07	0.20	0.13	0.28*	0.17	0.37**	0.11	0.46**	0.30*	−0.06	–	
13. Morpheme judgment	0.46**	−0.13	0.18	0.21	0.01	0.04	0.15	0.17	0.16	0.40**	−0.08	0.13	–
14. Homophonic judgment	0.73**	0.04	0.21	0.24	0.14	−0.02	0.27*	0.20	0.30*	0.36**	−0.05	0.43**	0.59**

* *p* < 0.05, ** *p* < 0.01.

In contrast, other domain-general abilities including spatial working memory, inhibitory control, visual attention, and non-verbal reasoning did not show significant differences between the two learner groups, all *p* > 0.05. This appears inconsistent with some previous studies in alphabetic L2 contexts, where executive functions and visuospatial memory have been shown to predict aspects of L2 achievement (Dörnyei and Skehan, 2003; Swanson and Berninger, 1995). There might be several reasons to explain this. First, prior studies often involved English or other alphabetic languages, where orthographic language relies more heavily on visual-phonological integration. In Chinese, the dominant role of phonological and morpheme processing relied on verbal working memory (Juffs and Harrington, 2011; Linck et al., 2014; Vasylets and Marín, 2021). Second, while spatial memory and visual attention are relevant for distinguishing character forms, they may not be limiting factors once learners reach a basic threshold of visual decoding ability (Tan et al., 2005). Lastly, inhibitory control and fluid reasoning may influence higher-order comprehension or task management but do not directly support the character-level processing critical in the early to intermediate stages of Chinese L2 acquisition (Linck et al., 2014; Vasylets and Marín, 2021). These findings converge with prior research emphasizing the important role of VWM in L2 learning (Gathercole and Baddeley, 1993; Kormos, 2023), particularly in morphologically rich or non-alphabetic systems.

### Morphological awareness as a core domain-specific cognitive factor in Chinese L2 learning

Analyses of domain-specific skills revealed that L2 learners Low-achieving Chinese L2 learners exhibited significantly poorer performance on all measures of morphological awareness compared to high-achieving learners. Specifically, significant differences were found in the morpheme judgment task (high-achieving: *M* = 0.92, SD = 0.07; low-achieving: *M* = 0.81, SD = 0.14; *t* = 3.79, *p* < 0.001) and the homophone judgment task (high-achieving: *M* = 0.88, SD = 0.08; low-achieving: *M* = 0.67, SD = 0.11; *t* = 8.73, *p* < 0.001). These findings align with existing research demonstrating that morphological awareness plays a fundamental role in supporting vocabulary acquisition and reading comprehension in Chinese L2 learners (Zhang et al., 2021). However, the difference in the nonword identification task was not statistically significant, *t* = 1.24, *p* > 0.05. These findings align with existing research demonstrating that morphological awareness plays a fundamental role in supporting vocabulary acquisition and reading comprehension in Chinese L2 learners.

As Chinese characters often encode meaning through embedded morphemes rather than through alphabetic sequences, the ability to recognize and manipulate morphological units is critical for efficient language processing in Chinese. Unlike alphabetic languages, where phonological decoding is typically the foundation for early literacy, Chinese requires learners to integrate morphological, semantic, and orthographic knowledge simultaneously. Morphological awareness enables learners to parse characters into meaningful morphemes, facilitating both decoding and inferencing during reading (Zhang et al., 2019). In nonword identification tasks, participants apply morphological rules to novel combinations, while semantic assessments rely on understanding morphemic meanings in context. In contrast, homophone judgment reflects learners’ sensitivity to morphological cues when multiple characters share the same phonological form but differ in meaning and written form. Underperformance in any of these areas could be detrimental to the development of character recognition, sentence-level comprehension, and vocabulary expansion. That might be why low-achieving Chinese L2 learners showed lower performance in Chinese achievement.

Taken together, these findings highlight the importance of integrating morphological training into L2 Chinese instruction, especially for learners who exhibit persistent reading and vocabulary difficulties. Explicit instruction in recognizing morphemes, differentiating homophones, and understanding character formation can enhance learners’ ability to construct semantic networks and decode unfamiliar words (Chow, 2018; Zhang and Roberts, 2019). Moreover, morphological awareness supports not only word-level recognition but also higher-level comprehension processes, making it a valuable target for interventions aimed at reducing achievement gaps. Supporting learners’ morphological development may thus be a key to unlocking more effective and inclusive Chinese L2 learning pathways.

### Theoretical and practical implications

The clear performance gap between high- and low-achieving Chinese L2 learners in verbal working memory and morphological awareness tasks highlights the differentiated cognitive demands of mastering Chinese as a second language. These results suggest that Chinese L2 learning is not uniformly constrained by general cognitive abilities but is instead shaped by specific processing demands, which particularly involved the temporary storage and manipulation of verbal information to identify morphemic units in characters.

This pattern reinforces the importance of distinguishing between domain-general and domain-specific cognitive factors in second language research. The salience of verbal working memory across tasks indicates that efficient temporary storage of phonological and lexical information is critical for processing morphosyllabic script. At the same time, the consistent underperformance of low-achieving learners in morphological awareness tasks points to this skill as a key bottleneck in vocabulary acquisition and reading comprehension. These findings refined existing models of Chinese L2 acquisition by showing how specific cognitive limitations directly contribute to persistent learning underperformance.

While our study did not directly test the causal impact of morphological awareness training, the performance gap between groups across all morphological tasks suggests that this ability is closely tied to broader language proficiency. Morphological tasks require skills directly transferable to reading and vocabulary learning. Therefore, we proposed that morphological awareness are not merely associated with low achievement, but are likely to impede key learning processes, making them a rational target for pedagogical intervention.

In practical terms, morphological awareness can be strengthened through explicit instruction that helps learners identify semantic radicals, analyze character formation rules, and apply morpheme-based inference strategies when encountering unfamiliar characters. For example, teachers can guide students to group characters by shared morphemes, practice decomposing compound characters into their functional components, and compare near-homophones in context to reinforce form-meaning mappings. These approaches help learners internalize the morphological system of Chinese, thereby improving their decoding efficiency and semantic access.

In addition to the interventions for low-achieving Chinese L2 learners, the strengths of high-achieving Chinese L2 learners should also be leveraged to further enhance their learning outcomes. For instance, high-achieving learners could be encouraged to engage in more complex cognitive tasks such as advanced character recognition exercises and sentence processing activities. These tasks can deepen their cognitive engagement with the language and further improve their proficiency. Additionally, high achievers could be encouraged to mentor low-achieving peers, creating a collaborative learning environment that benefits both groups. These strategies can help capitalize on the existing strengths of high achievers to foster even greater language learning achievements.

## Limitations

Despite the meaningful findings, several limitations of the present study should be acknowledged. First, this study focused on reading-related cognitive skills (e.g., working memory for character recognition, and morphological awareness) and did not assess other language domains, such as speaking, listening, or writing. Future research could expand on this by including other language domains for a more comprehensive understanding of the cognitive mechanisms involved in L2 acquisition. Second, the participant sample consisted of university students with relatively low Chinese proficiency, which may not represent learners at other developmental stages or proficiency levels. Third, although we examined cognitive variables such as working memory and morphological awareness, important non-cognitive factors including learner motivation, language exposure outside the classroom, and prior language learning experience were not assessed. Future research should adopt a more comprehensive framework that incorporates both cognitive and affective factors and investigates multiple language domains across diverse learner profiles.

## Data Availability

The original contributions presented in the study are included in the article/supplementary material, further inquiries can be directed to the corresponding author.

## Footnotes

^1^ www.dweipsy.com/lattice
